# Attitudes of the Brazilian Population Toward Organ Donation

**DOI:** 10.1016/j.ekir.2022.09.009

**Published:** 2022-09-15

**Authors:** Paulo Lisboa Bittencourt, Liana Codes, Elodie Bomfim Hyppolito, Heloisa Furia Cesar, José A. Moura-Neto, Maria Lucia Gomes Ferraz

**Affiliations:** 1 Hospital Português, Salvador, Bahia, Brazil; 2 Escola Bahiana de Medicina e Saúde Pública, Salvador, Bahia, Brazil; 3 Universidade de Fortaleza, Ceará, Brazil; 4 Serviço de Transplante de Fígado, Universidade Federal do Ceará, Ceará, Brazil; 5 Instituto Datafolha, São Paulo, Brazil; 6 Disciplina de Gastroenterologia, Universidade Federal de São Paulo, São Paulo, São Paulo, Brazil

**Keywords:** family refusal, organ donation, transplantation

## Introduction

Brazil ranks second worldwide in gross numbers of deceased donors for kidney and liver transplantation, but scores much lower in organ transplantation per million people, when compared to other countries.^1^ Therefore, more than 45,000 patients are currently on the waiting list for organ transplants in Brazil.^2^ In this country, transplantation activity was also shown to vary sharply.^2^ In this regard, organ transplantation is much more developed in the South and Southeast regions of Brazil when compared to the poorer parts of country due to higher donation rates, better organ procurement, as well as increased public education and awareness.^3^ Those aforementioned regions cover only 19% of the land mass but accounts for 56% of the population and 71% of the Brazilian Gross Domestic Product. Most of the liver and kidney transplantation centers are also concentrated in those 2 geographic regions. All aspects related to organ donation, procurement, and transplantation in Brazil are funded by the National “Unified” Public Health System.

One of the major barriers to increase transplantation activity in Brazil is a low organ donation rate,^3^ particularly due to an unacceptably high family refusal that approaches 40%.^1^^,^^2^ Deceased organ donation in Brazil requires consent from the donor family. Transition to an opt-out system, in which consent for donation is presumed, was attempted in the country in 1977. However, it has not succeeded due to its low acceptance by the general population with a consequent detrimental effect on organ donation.^2^^,^^4^ Since then, little is known about the awareness of the Brazilian population toward postmortem organ donation (PMOD). In order to investigate the perception and attitudes of the Brazilian population toward PMOD, a cross-sectional survey sponsored by the Brazilian Liver Institute was requested of the DataFolha Research Institute. The present manuscript describes the major findings of this survey.

## Results

**Figure 1:**
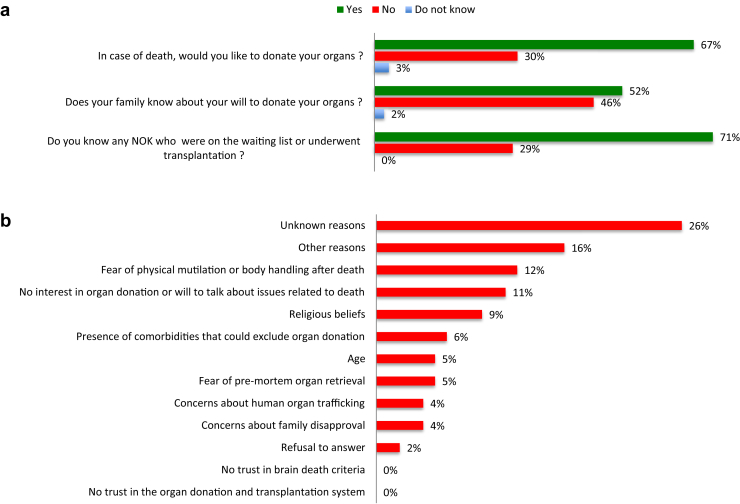
Answers to questions (a) related to attitudes regarding PMOD and (b) reasons against PMOD. Q1: In case of death, would you like to donate your organs?; Q2: If your decision is not to become an organ donor in case of death, for which reasons below you don’t want to donate your organs?; Q3 If your decision is to become an organ donor in case of death, does your family know about your will to donate your organs?; Q4: Do you know any relative or friend who underwent organ transplantation or were on the waiting list for transplantation?. PMOD, postmortem organ donation.

Using a cross-sectional survey approach (see Supplementary Methods in the Supplementary Material), 1976 participants (1050 women, mean age 44 years) from 129 municipalities from all Brazilian regions were interviewed. The demographic features of those subjects are depicted in Table 1 and are representative of the Brazilian population over 18 years of age. A great part of the subjects were middle-aged women from the Southeast, which is the most populated region of Brazil. Most of them lived in small municipalities in the countryside. Education level was up to high school in more than three-fourths of the subjects. Seventy percent of them were part of the economically active population. Most of them were from C or D/E socioeconomic classes and the mean household income of all participants was equivalent to 676 United States dollars. Seventy percent of them claimed to have children. The responses in respect to attitudes toward organ donation are summarized in Figure 1.

**Table 1:** Demographic data of all participants

Variables	All participants (*n* = 1976)		
	Unweighted	Weighted (%)	
Gender			
Female	1050	1056	(53)
Male	926	920	(47)
Age (yr)			
18–24	299	279	(14)
25–34	401	389	(20)
35–44	405	401	(20)
45–59	485	497	(25)
60 or more	386	411	(21)
Geographical region			
South	295	299	(15)
Southeast	844	863	(44)
Middle West	162	511	(26)
Northeast	520	153	(8)
North	155	150	(8)
Education level			
Elementary school	622	669	(34)
High school	877	873	(44)
Higher education	477	434	(22)
Socioeconomic class			
A/B	530	472	(24)
C	964	937	(47)
D/E	482	566	(29)
Place of living			
Metropolitan area of state capitals	870	824	(42)
Countryside small cities	1106	1152	(58)
Occupation			
EAP	1400	1378	(70)
Non-EAP	576	598	(30)
Children			
Yes	1362	1385	(70)
No	614	591	(30)

EAP, economically active population.

Among the subjects, 67% declared to agree to PMOD, 30% were not willing to donate their organs, and 3% were undecided (answered “do not know”). Consent for PMOD was more common in subjects younger than 44 years. Even though the willingness to donate their organs among those responders aged between 18 and 24 years seems to be higher, the difference was not significant when compared to the group between 24 and 44 years of age. Willingness to PMOD was more common among subjects living in metropolitan areas of state capitals and among individuals who were part of the economically active population or those who have no children (Supplementary Table S1 and Figure S1).

In general, participants with higher level of education or with higher household income declared more often to be organ donors. Consent for PMOD was similar when subjects from Southeast were compared to their counterparts from the South or Middle West region, but a lower willingness to donate was observed in the North and Northeast regions when compared to other regions (Supplementary Table S1 and Figure S1).

More than half of those participants willing to donate their organs after death have not informed their next of kin (NOK) about the decision. Most of the subjects who have shared their decision with their NOK were female. Those living in the South or Southwest, when compared Northeast region, and those living in metropolitan areas of state capitals or who were part of the economically active population also informed their NOK more often their decision of PMOD (Supplementary Table S2 and Figure S1).

Approximately, one-third of the responders declared having acquaintance with a transplant patient or someone who is on the waiting list for organ transplant. Less than often, these participants were between 18 to 24 years old or from class D/E. They were also more frequently from the Southeast (Supplementary Table S3) and more prone to donate their organs in case of death (33% vs. 19% of those who have no acquaintance with a transplant patient or someone on the waiting list for organ transplant, *P* < 0.05).

About half of the subjects who were not willing to donate their organs after death either do not know their reasons (26%), are afraid of body mutilation or handling after death (12%), or have no interest in organ donation or talking about death (11%) (Figure 1).

## Discussion

There is much more data in medical literature concerning factors associated with family refusal for organ donation^5^ than about perception and attitudes of the general population about PMOD.^6^^,^^7^ Willingness to donate organs after death varied sharply from 39% to 85% in reports from different countries^,^^8^^,^^9^ S1–S6 and in distinct population groups,S7–S11 due to several factors, including racial and ethnic backgrounds, religious beliefs, ethical issues, as well as lack of understanding about the donation process, particularly brain death criteria.^S12–S14^ Even in some of those countries who have adopted an opt-out system,^6^ family assent is taken into consideration. One of the major reasons for NOK to dissent from organ donation is the lack of previous awareness about the donors’ will, especially when it has not been appropriately registered.^5^^,^S12–S14 Because few studies have been reported covering representative portions of the general population,S2^,^S4^,^S7 this study ascertains the willingness of the Brazilian population to donate organs after death, its main demographic determinants, and whether or not the participants have informed their NOK about their decision. Similar to other reports from western^,^^9^^,^S2,S3,S7 and some Asian countriesS1, approximately two-thirds of Brazilians declared to be organ donors when questioned. In accordance with some^,^^9^^,^S1,S3,S7 findings but not all^S4^ reports, willingness to donate was associated with younger age and living in the Southeast and in metropolitan areas of state capitals. This may be due to better education and awareness but also to a higher acquaintance with people who benefited from organ donation, because most of the transplantation activity in Brazil occurs in the Southeast or in bigger cities outside of this region. Not surprisingly, having acquaintance with a transplant patient or someone on the waiting list was also associated with a higher willingness to donate in the present study. Despite the high willingness to donate organs observed in Brazilians, only half of them have shared their will to an NOK or friends. Those findings were also reported previously in Brazil^9^ and elsewhereS4 and underscoring the fact that people usually neglect to discuss topics related to death and dying.

In conclusion, Brazil has the largest public organ transplant program in the world, with an equitable and fair organ procurement and allocation system. However, despite the absolute numbers, there is still the need to increase the rate of transplants per million inhabitants. Though most Brazilians are willing to donate their organs, surprisingly only half of them have spoken to an NOK about their decision, making family consent to donation difficult in a country where donor registration is not required. It is time to better understand the scenario in order to change this reality. Public health policies are urgently needed in Brazil to raise awareness among the general population and, consequently, increase the willingness to donate organs after death.

## Disclosure

All the authors declared no competing interests.

### Funding

This work was supported by Brazilian Liver Institute.

## Supplementary Material

**Supplementary Methods.**

**Supplementary References.**

**Figure S1.** Multiple comparisons using SPSS.

**Table S1.** Willingness to donate organs after death according to demographics.

**Table S2.** Awareness of next of kin about decision to donate organs after death according to demographics.

**Table S3.** Acquaintance to someone transplanted or in the waiting list for transplantation according to demographics.

**Supplementary Bibliography.**

**STROBE Statement.**
