# Supplementary material for: Attitudes of the Brazilian Population Toward Organ Donation
Source: Kidney Int Rep. 2022 Sep 15;7(12):2737–40. doi: 10.1016/j.ekir.2022.09.009 (PMC9727522; doi:10.1016/j.ekir.2022.09.009)
Supplement: Supplementary File (PDF) [file mmc1.pdf]

Supplementary Methods

Patients and Methods:

The Brazilian Liver Institute requested to DataFolha Research Institute in August 2021 a cross-sectional survey in a sample representative of the Brazilian population older than 18years. As previously described,[S15-S18] the sample design was based on data from National Household Sample Survey (PNAD) 2019[S19] to be representative of the Brazilian population older than 18 years of age, including both genders and all socioeconomic strata and education levels. The study was conducted between August 2nd to August 7th 2021, accepting at most one sampling error of $\pm$ 2 percentage points and considering a 95% confidence interval. For 0.5% or lower estimates, zero was assumed. Sample bases lower than 30 cases were not considered for statistical analysis. The questions were divided in two blocks comprised by socioeconomic and demographic variables and specific overlapping questions regarding the research subject.

Socioeconomic and demographic variables included were age, gender, the geographic region in Brazil where data were collected (North, Northeast, Southeast, Middle West or South), place of living (metropolitan areas of state capitals or small countryside towns), education level (up to elementary school, up to high school and higher education), having children (yes or no), part of the economically active population (EAP) (yes or no) and social class according to average household income per month in US dollars (USD)[S20]: A/B, above 2286 USD; C, between 914 and 2286 USD and D/E, below 914 USD. US Dollar values were based in current rates as of January 2022.

The questions (Q) and possible answers (in parentheses) regarding the subject of the study were:

Q1: In case of death, would you like to donate your organs? (yes or no)

Q2: If your decision is not to become an organ donor in case of death, for which reasons below you don't want to donate your organs? (Religious beliefs, age; no interest in organ donation or will to talk about issues related to death; fear of physical mutilation after death, presence of comorbidities that could exclude organ donation, fear of body handling after death, fear of premortem organ retrieval, concerns about human organ trafficking, concerns about family disapproval, no trust in brain death criteria, no trust in the organ donation and transplantation system and other reasons)

Q3: If your decision is to become an organ donor in case of death, does your family know about your will to donate your organs? (yes or no)

Q4: Do you know any relative or friend who underwent organ transplantation or were on the waiting list for transplantation? (yes or no)

The possible answers to Q2 were elaborated based on the responses obtained in a pilot test performed in 17 subjects to assess how long it would take to answer both questionnaires, potential inconsistencies and most common responses.

The average length of each interview was 15 minutes. They were randomly carried outface-to-face by non-healthcare workers hired by the Datafolha Research Institute in public places using a tablet. Informed consent was obtained from all participants. They were also informed about the topic of the survey as well as its importance to current knowledge and public health policies. All methods were carried out following relevant guidelines and regulations. Informed consent was obtained from all subjects before each interview.

Statistical analysis: In order to assure that our sample was representative of the Brazilian population over 18, the data were weighted by demographics such as geographic region, gender as perceived by the interviewer and age.[8]

Briefly, the sampling weight was performed in order to equalize the distribution of the sample with the distribution of the desired Brazilian population.

Univariate analysis was tested using $\chi 2$ test or the Fisher exact probability test when appropriate. *P* values $\leq$ 0.05 were considered to be significant. For multiple comparisons between groups, p values were adjusted according to Bonferroni correction method. Statistical analyses with weighted data were performed with the Statistical Package for Social Sciences (SPSS Inc.,Chicago, IL, USA), version 21.0 for Windows.

Figure S1. Multiple Comparisons using SPSS

		AGE (Years)					Socioeconomic class		
		18 to 24	25 to 34	35 to 44	45 to 59	60 or +	A/ B	C	D/ E
		(A)	(B)	(C)	(D)	(E)	(A)	(B)	(C)
declared to be potential donors		78,74	74,96	71,44	61,18	54,77	77,66	69,23	54,83
**Multiple comparasons TEST**		**D E**	**D E**	**D E**			**B C**	**C**	
bases	weighted	279	389	401	497	411	52	85	336
	unweighted	299	401	405	485	386	62	96	372

		AGE (Years)					Socioeconomic class		
		18 to 24	25 to 34	35 to 44	45 to 59	60 or +	A/ B	C	D/ E
		(A)	(B)	(C)	(D)	(E)	(A)	(B)	(C)
informed their families about the decision		45,64	58,06	59,95	53,35	39,88	67,21	52,24	34,58
**Multiple comparasons TEST**			**E**	**A E**	**E**		**BC**	**C**	
bases	weighted	219	292	286	304	225	368	651	311
	unweighted	236	302	292	299	217	412	666	268

		AGE (Years)					Socioeconomic class		
		18 to 24	25 to 34	35 to 44	45 to 59	60 or +	A/ B	C	D/ E
		(A)	(B)	(C)	(D)	(E)	(A)	(B)	(C)
have an acquaintance with a transplant patient or someone who is on the waiting list for organ transplant.		21,15	27,34	30,04	31,66	29,83	35,01	28,48	23,52
**Multiple comparasons TEST**					**A**		**B C**		
bases	weighted	279	389	401	497	411	472	937	566
	unweighted	299	401	405	485	386	530	964	482

		Geographical region					Education Level		
		South	South east	Middle West	Northe ast	North	Elementary School	High School	Higher Education
		(A)	(B)	(C)	(D)	(E)	(A)	(B)	(C)
declared to be potential donors		72,55	67,94	59,04	69,28	59,54	55,68	69,78	79,40
**Multiple comparasons TEST**		**C E**						**A**	**A B**
bases	weighted	863	299	511	153	150	669	873	434
	unweighted	844	295	520	162	155	622	877	477

		Geographical region					Education Level		
		South	South east	Middle West	Northe ast	North	Elementary School	High School	Higher Education
		(A)	(B)	(C)	(D)	(E)	(A)	(B)	(C)
informed their families about the decision		56,28	59,56	42,49	45,03	48,87	37,52	50,28	71,64
**Multiple comparasons TEST**		**C**	**C**					**A**	**A B**
bases	weighted	626	203	302	106	89	372	609	345
	unweighted	612	202	320	117	95	350	622	374

		Geographical region					Education Level		
		South	South east	Middle West	Northe ast	North	Elementary School	High School	Higher Education
		(A)	(B)	(C)	(D)	(E)	(A)	(B)	(C)
have an acquaintance with a transplant patient or someone who is on the waiting list for organ transplant.		32,30	24,61	26,18	25,69	26,73	25,00	28,29	34,85
**Multiple comparasons TEST**									**A B**
bases	weighted	863	299	511	153	150	669	873	434
	unweighted	844	295	520	162	155	622	877	477

Table S1. Willingness to donate organs after death according to demographics

Variables	All participants* (n=1976)				Test for equality of proportions		95% CI		
	Unweighted	Weighted (%)		Yes (%)	$\chi2$	*p*	SE	LL (%)	UL (%)
Gender					2.096	0.553			
Female	1050	1056	(53)	(68)			1.049	66	70
Male	926	920	(47)	(67)			1.058	65	69
Age (years)					80.619	<0.0001			
18 to 24	299	279	(14)	(79)			0.916	77	81
25 to 34	401	389	(20)	(75)			0.974	73	77
35 to 44	405	401	(20)	(71)			1.021	69	73
45 to 59	485	497	(25)	(61)			1.097	59	63
60 or more	386	411	(21)	(55)			1.119	53	57
Geographical region					37.772	<0.0001			
South	295	299	(15)	(68)			1.049	66	70
Southeast	844	863	(44)	(73)			0.999	71	75
Middle West	162	511	(26)	(69)			1.040	67	71
Northeast	520	153	(8)	(59)			1.106	57	61
North	155	150	(8)	(60)			1.102	58	62
Education Level					81.589	<0.0001			
Elementary School	622	669	(34)	(56)			1.117	54	58
High School	877	873	(44)	(70)			1.031	68	72
Higher Education	477	434	(22)	(79)			0.916	77	81
Socioeconomic class					80.524	<0.0001			
A/B	530	472	(24)	(78)			0.932	76	80
C	964	937	(47)	(69)			1.040	67	71
D/E	482	566	(29)	(55)			1.119	53	57

Place of Living					35.038	<0.0001			
Metropolitan area of statecapitals	870	824	(42)	(74)			0.987	72	76
Countryside small cities	1106	1152	(58)	(62)			1.092	60	64
Occupation					30.070	<0.0001			
EAP	1400	1378	(70)	(71)			1.021	69	73
Non-EAP	576	598	(30)	(59)			1.106	57	61
Children					27.386	<0.0001			
Yes	1362	1385	(70)	(64)			1.080	62	66
No	614	591	(30)	(75)			0.974	73	77

* For multiple comparisons see Appendix I

CI, confidence interval; LL, lower limit; UL, upper limit; SE, standard error; EAP, economically active population.

Table S2. Awareness of next of kin about decision to donate organs after death according to demographics

Variables	Participants* (n=1346)			Test for equality of proportions			95% CI	
	Unweighted	Weighted (%)	Yes (%)	$\chi 2$	*p*	SE	LL (%)	UL (%)
Gender				5.330	0.070			
Female	625	613 (46)	(56)			1.117	54	58
Male	721	714 (54)	(51)			1.125	49	53
Age years				31.116	<0.0001			
18 to 24	236	219 (17)	(47)			1.123	45	49
25 to 34	302	292 (22)	(59)			1.106	57	61
35 to 44	292	286 (22)	(63)			1.086	61	65
45 to 59	299	304 (23)	(54)			1.121	52	56
60 or more	217	225 (17)	(42)			1.110	40	44
Geographical region				26.071	0.001			
South	612	626 (47)	(62)			1.092	60	64
Southeast	202	203 (15)	(58)			1.110	56	60
Middle West	320	302 (23)	(46)			1.121	44	48
Northeast	117	106 (8)	(44)			1.117	42	46
North	95	89 (7)	(49)			1.125	47	51
Education Level				86.744	0.000			
Elementary School	350	372 (28)	(39)			1.097	37	41
High School	622	609 (46)	(52)			1.124	50	54
Higher Education	374	345 (26)	(72)			1.010	70	74
Socioeconomic class				72.359	0.000			
A/B	412	367 (28)	(69)			1.040	67	71
C	666	649 (49)	(54)			1.121	52	56
D/E	268	310 (23)	(36)			1.080	34	38
Place of Living				5.516	0.063			

Metropolitan area of state capitals	645	608	(46)	(57)			1.114	55	59
Countryside small cities	701	718	(54)	(51)			1.125	49	53
Occupation					12.288	0,002			
EAP	1000	975	(73)	(56)			1.117	54	58
Non-EAP	346	351	(26)	(47)			1.123	45	49
Children					0.701	0.700			
Yes	884	882	(66)	(53)			1.123	51	55
No	462	444	(33)	(55)			1.119	53	57

* For multiple comparisons see Appendix I

CI, confidence interval; LL, lower limit; UL, upper limit; SE, standard error; EAP, economically active population

Table S3. Acquaintance to someone transplanted or in the waiting list for transplantation according to demographics

Variables	All participants (n=1976)				Test for equality of proportions			95% CI	
	Unweighted	Weighted (%)		Yes (%)	$\chi 2$	*p*	SE	LL (%)	UL (%)
Gender					0.400	0.819			
Female	1050	1056	(53)	(28)			1.010	26	30
Male	926	920	(47)	(29)			1.021	27	31
Age years					20.436	0.009			
18 to 24	299	279	(14)	(21)			0.916	19	23
25 to 34	401	389	(20)	(27)			0.999	25	29
35 to 44	405	401	(20)	(30)			1.031	28	32
45 to 59	485	497	(25)	(32)			1.049	30	34
60 or more	386	411	(21)	(30)			1.031	28	32
Geographical region					12.121	0.146			
South	295	299	(15)	(25)			0.974	23	27
Southeast	844	863	(44)	(32)			1.049	30	34
Middle West	162	511	(26)	(26)			0.987	24	28
Northeast	520	153	(8)	(26)			0.987	24	28
North	155	150	(8)	(27)			0.999	25	29
Education Level					13.237	0.010			
Elementary School	622	669	(34)	(25)			0.974	23	27
High School	877	873	(44)	(28)			1.010	26	30
Higher Education	477	434	(22)	(35)			1.073	33	37
Socioeconomic class					17.495	0.002			
A/B	530	472	(24)	(35)			1.073	33	37
C	964	937	(47)	(28)			1.010	26	30

D/E	482	566	(29)	(24)			0.961	22	26
Place of Living					5.673	0.059			
Metropolitan area of state capitals	870	824	(42)	(28)			1.010	26	30
Countryside small cities	1106	1152	(58)	(29)			1.021	27	31
Occupation					4.961	0.084			
EAP	1400	1378	(70)	(29)			1.021	27	31
Non-EAP	576	598	(30)	(27)			0.999	25	29
Children					2.054	0.358			
Yes	1362	1385	(70)	(29)			1.021	27	31
No	614	591	(30)	(27)			0.999	25	29

* For multiple comparisons see Appendix I

CI, confidence interval; LL, lower limit; UL, upper limit; SE, standard error; EAP, economically active population

Supplemental Bibliography

S1. Muthiah MD, Chua MSH, Griva K, et al. A Multiethnic Asian Perspective of Presumed Consent for Organ Donation: A Population-Based Perception Study. *Front Public Health*. 2021;9:712584. Published 2021 Oct 5. doi:10.3389/fpubh.2021.712584

S2. Decker O, Winter M, Brähler E, Beutel M. Between commodification and altruism: gender imbalance and attitudes towards organ donation. A representative survey of the German community. J Gender Stud, 17 (2008), pp.251-255

S3. Kobus G, Popławska W, Zbroch E, Małyszko J, Bachórzewska-Gajewska H, Małyszko J. Opinions of town residents on organ transplantation. Transplant Proc. 2014 Oct;46(8):2492-5. doi: 10.1016/j.transproceed.2014.09.020. PMID: 25380851.

S4. Webb G, Phillips N, Reddiford S, Neuberger J. Factors Affecting the Decision to Grant Consent for Organ Donation: A Survey of Adults in England. Transplantation. 2015 Jul;99(7):1396-402. doi: 10.1097/TP.0000000000000504. PMID: 25675195.

S5. Mohamed E, Guella A. Public awareness survey about organ donation and transplantation. Transplant Proc. 2013;45(10):3469-71. doi: 10.1016/j.transproceed.2013.08.095. PMID: 24314934.

S6.Tarzi M, Asaad M, Tarabishi, J., et al. Attitudes towards organ donation in Syria: a cross-sectional study. BMC medical ethics, 2020, 21(1), 1-10.

S7. 2019 National Survey of Organ Donation Attitudes and Practices: Report of Findings. Published February 2020 U.S. Department of Health and Human Services Health Resources and Services Administration Healthcare Systems Bureau Division of Transplantation. www.organdonor.gov

S8. Morgan M, Kenten C, Deedat S; Donate Programme Team. Attitudes to deceased organ donation and registration as a donor among minority ethnic groups in North America and the U.K.: a synthesis of quantitative and qualitative research. Ethn Health. 2013;18(4):367-90. doi: 10.1080/13557858.2012.752073. Epub 2012 Dec 19. PMID: 23249284.

S9. Davison SN, Jhangri GS. Knowledge and attitudes of Canadian First Nations people toward organ donation and transplantation: a quantitative and qualitative analysis. Am J Kidney Dis. 2014 Nov;64(5):781-9. doi: 10.1053/j.ajkd.2014.06.029. Epub 2014 Aug 27. PMID: 25172531.

S10. Bastami S, Matthes O, Krones T, et al. Systematic review of attitudes toward donation after cardiac death among healthcare providers and the general public. CritCareMed 2013;41:897–905.
S11. Tackmann E, Kurz P, Dettmer S. Attitudes and knowledge about post-mortem organ donation among medical students, trainee nurses and students of health sciences in Germany : A cross-sectional study. Anaesthesist. 2020 Nov;69(11):810-820. doi: 10.1007/s00101-020-00812-8.
S12. Da Silva IR, Frontera JA. Worldwide barriers to organ donation. JAMA Neurol. 2015 Jan;72(1):112-8. doi: 10.1001/jamaneurol.2014.3083. PMID: 25402335.
S13. Lewis A, Koukoura A, Tsianos GI, Gargavanis AA, Nielsen AA, Vassiliadis E. Organ donation in the US and Europe: The supply vs demand imbalance. Transplant Rev (Orlando). 2021 Apr;35(2):100585. doi: 10.1016/j.trre.2020.100585. Epub 2020 Oct 11. PMID: 33071161.
S14. Ralph A, Chapman JR, Gillis J, et al. Family perspectives on deceased organ donation: thematic synthesis of qualitative studies. Am J Transplant. 2014 Apr;14(4):923-35. doi: 10.1111/ajt.12660. Epub 2014 Mar 10. PMID: 24612855.
S15. Bittencourt LR., Santos-Silva R, Taddei JA, Andersen ML, Mello MT, Tufik S. Sleep complaints in the adult Brazilian population: A national survey based on screening questions. J Clin Sleep Med. 2009; 5: 459–463.
S16. Hirotsu C, Bittencourt LR, Garbuio S, Andersen M L, Tufik S. Sleep complaints in the Brazilian population: Impact of socioeconomic factors. Sleep Science. 2014; 73: 135142.
S17. Spizzirri G, Eufrásio R, Lima MCP, et al. Proportion of people identified as transgender and non-binary gender in Brazil. Sci Rep. 2021 Jan 26;11(1):2240. doi: 10.1038/s41598-021-81411-4. PMID: 33500432; PMCID: PMC7838397.
S18. Kish L. Survey Sampling. John Wiley, Sons Inc, New York 1965
S19. www.ibge.org.br
S20. Associação Brasileira de Empresas de Pesquisa. Critério de Classificação Econômica Brasil, http://www.abep.org/crite riobrasil 2018.

STROBE Statement. Checklist of items that should be included in reports of observational studies

	Item No	Recommendation	Page No
**Title and abstract**	1	(*a*) Indicate the study's design with a commonly used term in the title or the abstract	3
		(*b*) Provide in the abstract an informative and balanced summary of what was done and what was found	N/A
**Introduction**			
Background/rationale	2	Explain the scientific background and rationale for the investigation being reported	4
Objectives	3	State specific objectives, including any prespecified hypotheses	5
**Methods**			
Study design	4	Present key elements of study design early in the paper	5
Setting	5	Describe the setting, locations, and relevant dates, including periods of recruitment, exposure, follow-up, and data collection	5,6
Participants	6	(*a*) *Cohort study*—Give the eligibility criteria, and the sources and methods of selection of participants. Describe methods of follow-up<br>*Case-control study*—Give the eligibility criteria, and the sources and methods of case ascertainment and control selection. Give the rationale for the choice of cases and controls<br>*Cross-sectional study*—Give the eligibility criteria, and the sources and methods of selection of participants	5,6
		(*b*)*Cohort study*—For matched studies, give matching criteria and number of exposed and unexposed<br>*Case-control study*—For matched studies, give matching criteria and the number of controls per case	
Variables	7	Clearly define all outcomes, exposures, predictors, potential confounders, and effect modifiers. Give diagnostic criteria, if applicable	5
Data sources/measurement	8*	For each variable of interest, give sources of data and details of methods of assessment (measurement). Describe comparability of assessment methods if there is more than one group	6
Bias	9	Describe any efforts to address potential sources of bias	5,6,7
Study size	10	Explain how the study size was arrived at	5
Quantitative variables	11	Explain how quantitative variables were handled in the analyses. If applicable, describe which groupings were chosen and why	6

Statistical methods	12	(*a*) Describe all statistical methods, including those used to control for confounding	7
		(*b*) Describe any methods used to examine subgroups and interactions	5,6,7
		(*c*) Explain how missing data were addressed	N/A
		(*d*) *Cohort study*—If applicable, explain how loss to follow-up was addressed<br>*Case-control study*—If applicable, explain how matching of cases and controls was addressed<br>*Cross-sectional study*—If applicable, describe analytical methods taking account of sampling strategy	5,6,7
		(*e*) Describe any sensitivity analyses	7
**Results**			
Participants	13*	(a) Report numbers of individuals at each stage of study—eg numbers potentially eligible, examined for eligibility, confirmed eligible, included in the study, completing follow-up, and analysed	7
		(b) Give reasons for non-participation at each stage	N/A
		(c) Consider use of a flow diagram	N/A
Descriptive data	14*	(a) Give characteristics of study participants (eg demographic, clinical, social) and information on exposures and potential confounders	7
		(b) Indicate number of participants with missing data for each variable of interest	24
		(c) *Cohort study*—Summarise follow-up time (eg, average and total amount)	
Outcome data	15*	*Cohort study*—Report numbers of outcome events or summary measures over time	
		Case-control study—Report numbers in each exposure category, or summary measures of exposure	
		Cross-sectional study—Report numbers of outcome events or summary measures	8,9
Main results	16	(*a*) Give unadjusted estimates and, if applicable, confounder-adjusted estimates and their precision (eg, 95% confidence interval). Make clear which confounders were adjusted for and why they were included	8,9
		(*b*) Report category boundaries when continuous variables were categorized	18-24
		(*c*) If relevant, consider translating estimates of relative risk into absolute risk for a meaningful time period	N/A
Other analyses	17	Report other analyses done—eg analyses of subgroups and interactions, and sensitivity analyses	24
**Discussion**			
Key results	18	Summarise key results with reference to study objectives	10

Limitations	19	Discuss limitations of the study, taking into account sources of potential bias or imprecision. Discuss both direction and magnitude of any potential bias	10
Interpretation	20	Give a cautious overall interpretation of results considering objectives, limitations, multiplicity of analyses, results from similar studies, and other relevant evidence	9-11
Generalisability	21	Discuss the generalisability (external validity) of the study results	11
**Other information**			
Funding	22	Give the source of funding and the role of the funders for the present study and, if applicable, for the original study on which the present article is based	11

*Give information separately for cases and controls in case-control studies and, if applicable, for exposed and unexposed groups in cohort and cross-sectional studies.

Note: An Explanation and Elaboration article discusses each checklist item and gives methodological background and published examples of transparent reporting. The STROBE checklist is best used in conjunction with this article (freely available on the Web sites of PLoS Medicine at http://www.plosmedicine.org/, Annals of Internal Medicine at http://www.annals.org/, and Epidemiology at http://www.epidem.com/). Information on the STROBE Initiative is available at www.strobe-statement.org.
